# Night Shift Among Women: Is It Associated With Difficulty Conceiving a First Birth?

**DOI:** 10.3389/fpubh.2020.595943

**Published:** 2020-12-01

**Authors:** Renae C. Fernandez, Vivienne M. Moore, Jennifer L. Marino, Melissa J. Whitrow, Michael J. Davies

**Affiliations:** ^1^Adelaide Medical School, The University of Adelaide, Adelaide, SA, Australia; ^2^School of Public Health, The University of Adelaide, Adelaide, SA, Australia; ^3^Lifecourse and Intergenerational Health Research Group, Robinson Research Institute, Adelaide, SA, Australia; ^4^Fay Gale Centre for Research on Gender, Adelaide, SA, Australia; ^5^Department of Obstetrics and Gynaecology, University of Melbourne, Melbourne, VIC, Australia; ^6^Royal Women's Hospital, Melbourne, VIC, Australia; ^7^Centre for Adolescent Health, Murdoch Children's Research Institute, Melbourne, VIC, Australia

**Keywords:** assisted reproduction (ART), endometriosis, infertility, menstrual abnormality, shift work (MeSH), night shift work

## Abstract

**Background:** Asynchrony in circadian processes alters many physiological systems, including female reproduction. Thus, there are possible reproductive consequences of night shift work for women including menstrual irregularity, endometriosis, and prolonged time to conception. This study examined whether women who worked night shift were more likely than those who did not to require fertility treatment to conceive a first birth, whether they had specific infertility diagnoses, and if such relationships were age-specific.

**Methods:** In a retrospective data linkage study of 128,852 primiparous women, fertility treatment data were linked to the state perinatal registry for South Australia (1986–2002). Potential exposure to night shift work was assessed using a job-exposure matrix. First, the association between night shift work and fertility treatment was assessed among (1) all women, then (2) women in paid employment, using logistic regression. Interactions between age and shift work status were also examined. Secondly, among women who conceived with fertility treatment, we assessed associations between night shift work and type of infertility diagnosis. Potential confounders were considered in all analyses.

**Results:** Among women ≤35 years, night shift workers were more likely to require fertility treatment (all: OR = 1.40, 95% CI 1.19–1.64; in paid employment: OR = 1.27, 95% CI 1.08–1.50). There were no associations among women >35 years. Ethnicity, socioeconomic status and smoking did not affect these results. Among women who underwent fertility treatment, night shift workers were more likely than day workers to have menstrual irregularity (OR = 1.42, 95% CI 1.05–1.91) or endometriosis (OR = 1.34, 95% CI 1.00–1.80).

**Conclusions:** Night shift work may contribute to increased need for fertility treatment in younger women. This increased risk may reflect young women's vulnerability in terms of poor tolerance of night shift work, and/or lack of control and choice about shift schedule.

## Introduction

The nature of paid work and the workforce in Western societies is changing, with manual laboring jobs declining and demand for workers in the service and care industries increasing (1). One implication of this is increased non-standard and flexible working time arrangements (2). Such changes in work arrangements disproportionately affect women, who predominate in the growth industries (3).

Night shift work, in particular, may interfere with the lives and reproductive health of women. Quantity and quality of sleep can be affected and the circadian rhythm, the 24-h biological cycle that regulates sleep and wakefulness, can be disrupted (4). Asynchrony in circadian processes alters many physiological systems, including female reproduction (5, 6). Fixed night shift and rotating schedules that include night shift are thought to have the greatest impact (4).

Possible reproductive consequences of night shift work for women include menstrual irregularity (7), endometriosis (8, 9), and prolonged time to conception (10). To our knowledge, no study has investigated the potential relationship between night shift work and the requirement for reproductive assistance (fertility treatment) to conceive. Australia provides an ideal context in which to study this relationship since fertility treatment services are more accessible in Australia than in most other countries. In particular, fertility treatments and associated pharmaceutical costs have been subsidized since as early as 1990 (11), and there are no restrictions to access based on age, number of treatment cycles or existing family size (12, 13).

The aim of this study was to investigate whether primiparous women employed in occupations potentially involving night shift work were more likely than women in occupations not involving night shift work to require fertility treatment, and if so, to characterize the type of infertility diagnoses. We considered the role of age to explicitly address the circumstances that: night shift work is more commonly undertaken by younger women, including within occupations such as nursing where more senior positions typically entail (administrative) day work; access to fertility treatment increases with age, as women are increasingly in a position to bear associated costs (financial, time, relationship strain); the age-related decline in women's fertility changes the demographic and health profiles of women seeking treatment.

## Materials and Methods

### Data Sources and Study Population

**Figure 1:**
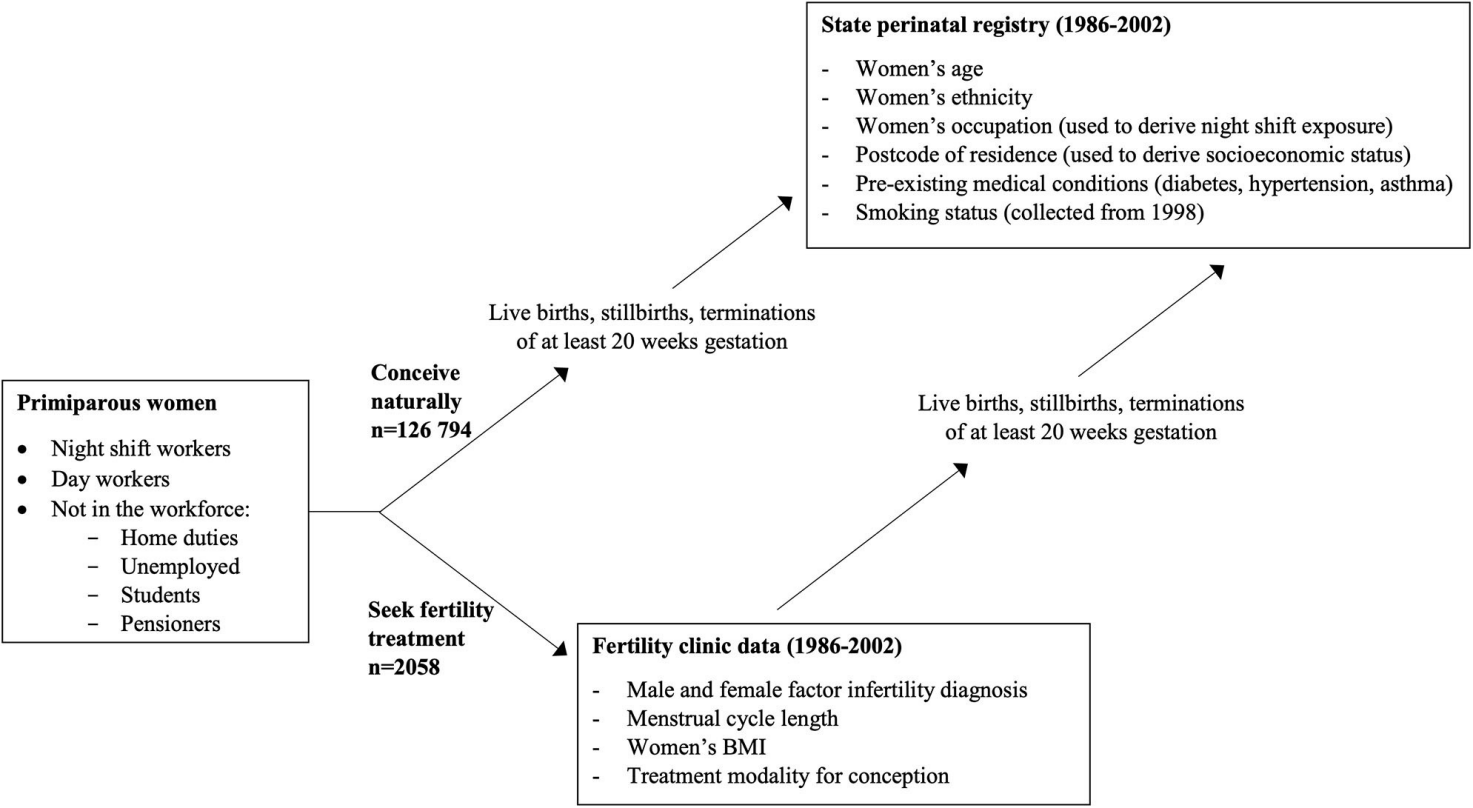
Sources of study data and key variables.

As described previously (14), the cohort for this study was retrospectively assembled by linking population-wide data from the South Australian perinatal registry (for the period January 1986 to December 2002) to data relating to patients undergoing assessment and treatment for infertility. Data sets and key variables are depicted in Figure 1.

### Night Shift Work

The perinatal registry includes a woman's usual occupation prior to and/or during pregnancy (15), coded according to the Australian Standard Classification of Occupations First Edition (16). To assess exposure to night shift work, a shift work job-exposure matrix (JEM) was applied. Job-exposure matrices provide a cross-classification of job codes/titles and the probability of occupational exposure (17). A detailed description of the specific shift work JEM, including its validation, has been published elsewhere (18). The JEM assigns each occupation a probability of exposure to light at night, phase shift, sleep disturbances, and other factors (19). For the present study, exposure to light at night was selected as an indicator of night and rotating shift work that includes nights. Exposure to light at night is a key contributor to circadian disruption and altered melatonin secretion, both of which have been associated with several adverse health outcomes (20). Occupations with exposure to light at night were those in which at least 30% of workers reported exposure, an optimal threshold as determined in previous studies (21). Those labeled “night shift workers” were a member of those occupations. Those without this were assumed to be day workers.

### Definition of Variables

Details of infertility diagnosis and fertility treatment were obtained from infertility clinic records (Figure 1). Women were considered to have required fertility treatment if they conceived by any form of clinic-based fertility treatment including ovulation induction, intrauterine insemination, *in vitro* fertilization (IVF), and intracytoplasmic sperm injection (ICSI). Births conceived to couples with male-factor infertility as the primary infertility diagnosis (*n* = 1,437) were excluded from all analyses to ensure that these women were not incorrectly classified (with their independent requirement for fertility treatment frequently unclear).

Among women who required fertility treatment to conceive, infertility diagnosis was categorized as: ovulatory dysfunction (including polycystic ovary syndrome), tubal blockage/problem, endometriosis (usually after visual inspection of the pelvic cavity), menstrual irregularity, and unexplained female-factor infertility (22). Menstrual irregularity was derived from self-reported usual cycle length at the beginning of treatment cycle (<24 days or >32 days, or “irregular” in place of length). Apart from unexplained female-factor infertility, women could be assigned more than one diagnosis category.

Demographic, lifestyle, and health characteristics for all primiparous women were obtained from the perinatal registry. Women's age at delivery (5-years age groups) enabled comparison with other women who did not access treatment (for whom age at conception is not a data item). Other covariates considered were ethnicity (Caucasian vs. non-Caucasian) and socio-economic status based on the level of disadvantage of a woman's area of residence (derived from the Socio-Economic Indices for Areas developed by the Australian government) (23). A small number of women for whom postcode, and therefore, socioeconomic quartile was missing (*n* = 362, 0.3%) were excluded from analyses involving this variable. Pre-pregnancy medical conditions considered were diabetes, hypertension and asthma. Smoking status was routinely recorded on the perinatal record from 1998. Body mass index (BMI) was not recorded in the perinatal dataset but was available for around three quarters of fertility treatment patients.

### Statistical Analysis

The study population was restricted to primiparous women in order to increase the likelihood that participants were employed in their designated usual occupation around the time of conception and to reduce potential bias associated with the ‘infertile worker’ effect (24, 25). The infertile worker effect is observed in occupational studies when women who begin family formation earlier and/or conceive quickly leave the workforce, artificially creating the appearance that women who remain, and are therefore available for study, are more likely to be childless. This is an important consideration as half of Australian women (53%) reduce participation in the workforce after giving birth. While most return to work within 2 years, this is usually (84%) part-time, which would affect night shift work exposure (26).

The proportions of women in occupational subgroups, classified by potential night shift exposure, were examined. The proportions conceiving with fertility treatment were calculated for these subgroups and also for those not in the paid workforce (home duties, students, unemployed, pensioners). Categorical variables were summarized using frequencies and percentages, and continuous variables using means and standard deviations. Demographic, lifestyle, and health characteristics were compared between those who did and did not work night shift, and between those who did and did not use fertility treatment, using *t*-tests for continuous variables, Fisher's exact tests for binary variables, and chi-squared tests for categorical variables.

Relationships between shift work and fertility treatment were assessed using multivariate logistic regression. Characteristics which were related to shift work or fertility treatment in bivariate analyses were included in multivariate analyses. Effect modification by age was assessed with an interaction term. Age at delivery was dichotomized as ≤35 or >35 years for the purposes of the interaction analysis, consistent with the inflection point for the age at which decline in female fertility is observed (27, 28). Two reference groups were used. First, night shift working women were compared with all other women not exposed to shift work, including those not in the paid workforce. Second, the comparison group was restricted to day workers, that is, women in paid employment who were not classified as potentially exposed to night shift.

A high proportion of female shift workers in Australia are employed as nurses (29), which may introduce bias due to nurses' familiarity with health and the health care system possibly influencing their engagement with treatment for infertility. Therefore, a sensitivity analysis was performed in which women employed as nurses were excluded. Smoking was a potential confounding variable, but as smoking was recorded for only part of the study period, this could only be investigated in a sensitivity analysis using a restricted dataset containing this variable, i.e., from 1998 onwards.

For women whose first birth was conceived with fertility treatment, infertility diagnoses were tabulated according to night shift exposure. Associations were investigated in detail using logistic regression and consideration of potential confounding factors as above. Sensitivity analyses for smoking were undertaken as previously and additional sensitivity analyses for BMI were performed.

All hypothesis tests were two-sided and *p* < 0.05 were considered statistically significant. Data analysis was performed using Stata v.14. (StataCorp, College Station, Texas, USA).

### Ethical Approval

The study was approved by the ethics committees of the South Australian Department of Health, the University of Adelaide, and Flinders University. Individual patient consent was not required by the ethics committees.

## Results

Of the 128,852 primiparous women who gave birth during the study period, 11,000 (8.5%) were employed in occupations that were likely to have involved night shift (Table 1). The majority of potential night shift workers (72.7%) were registered or enrolled nurses (i.e., degree or diploma qualification). The largest occupational groups among presumed day workers were clerks and sales assistants, followed by teachers. One in five women were unemployed or engaged in home duties.

**Table 1:** Births to primiparous women 1986–2002 by employment status, occupation and mode of conception.

**Employment status**	**All**		**Proportion of occupation subcategory**	**Conceived with fertility treatment^c^**	
	***N***	**%**	**%**	***N***	**%**
All women	128,852	100.0	-	2,058	1.6
Night shift occupations	11,000	8.5	100.0	243	2.2
Registered nurses	6,405	5.0	58.2	157	2.5
Other personal service workers (e.g., croupier)	1,818	1.4	16.5	32	1.8
Enrolled nurses	1,596	1.2	14.5	31	1.9
Police	383	0.3	3.5	11	2.9
Radiographers	209	0.2	1.9	5	2.4
Food processing machine operators	148	0.1	1.3	1	0.7
Actors and related professionals	103	0.1	0.9	0	0.0
Other shift working occupations^a^	84	0.1	0.8	2	2.4
Guards & security officers	75	0.1	0.7	2	2.7
Photographic products machine operators	65	0.1	0.6	2	3.1
Securities & finance dealers	62	0.05	0.6	0	0.0
Metal fitters & machinists	52	0.04	0.5	0	0.0
Day work occupations	84,991	66.0	100.0	1,514	1.8
Other clerks	13,071	10.1	15.4	248	1.9
Sales assistants	10,318	8.0	12.1	109	1.1
Teachers^b^	4,573	3.5	5.4	126	2.8
All other day working occupations	57,029	42.8	67.1	1,031	1.8
Not in paid employment	30,147	25.5	100.0	301	0.9
Home duties	14,419	11.2	47.8	240	1.7
Unemployed	11,835	9.2	39.3	32	0.3
Students	3,416	2.7	11.3	14	0.4
Pensioners	477	0.4	1.5	3	0.6
Unknown occupation	2,714	2.1	100.0	12	0.4

a *Data combined for shift working occupations where n < 30 (air transport operating support workers, prison officers, production recording clerks, other stationary plant operators, fabric production machine operators)*.

b *Includes pre-primary, primary, secondary and extra-systematic teachers, but not tertiary teachers*.

c *Couples who accessed fertility treatment for any diagnosis other than male factor infertility only*.

Overall, 1.6% of first births were conceived with fertility treatment (Table 1). For night shift workers the proportion was 2.2%. Use of fertility treatment for conception was least common among those not in paid employment: these women accounted for only 14.5% of births conceived with fertility treatment, compared with 25.9% of naturally conceived births.

As expected, maternal age, ethnicity, socioeconomic status and smoking were associated with conception using fertility treatment. Night shift workers tended to be older, Caucasian, and to live in the most economically advantaged areas compared to day workers (Table 2). Although smoking was less common among night shift workers overall, smoking prevalence for occupations involving night shift work was highly variable: for example, 4.9% for registered nurses, 12.2% for enrolled nurses, and 26.7% for guards and security officers. Socioeconomic status also varied across night shift working occupations; the proportion of women in the lowest socioeconomic quartile was 13.7% for registered nurses, 17.4% for enrolled nurses and 24.0% for guards and security officers. There was little difference in the overall prevalence of pre-pregnancy medical conditions among women in paid employment when stratified by exposure to night shift work.

**Table 2:** Demographic, health and lifestyle characteristics of primiparous women giving birth 1986–2002.

								**Mode of conception**				
**Characteristic**	**Night shift workers (*****N*** **= 11,000)**		**Day workers (*****N*** **= 84,991)**		**Night shift vs. day workers**	**Not in paid employment (*****N*** **= 32,861)**		**Fertility treatment conceptions (*****N*** **= 2,058)**		**Natural conceptions (*****N*** **= 126,794)**		**Treatment vs. Natural**
	***N***	**%**	**N**	**%**	***P*-value**	***N***	**%**	***N***	**%**	***N***	**%**	***P*-value**
**AGE (YEARS)**												
<30	7,139	64.9	60,185	70.8	<0.001	28,717	87.4	579	28.1	95,462	75.3	<0.001
30–34	2,951	26.8	19,057	22.4		3,059	9.3	909	44.2	24,158	19.1	
35–39	797	7.3	5,027	5.9		913	2.8	474	23.0	6,263	4.9	
≥40	113	1.0	720	0.8		169	0.5	96	4.7	906	0.7	
**ETHNICITY**												
Caucasian	10,716	97.4	81,581	96.0	<0.001	28,369	86.3	1,978	96.1	118,688	93.6	<0.001
Non-Caucasian	284	2.6	3,410	4.0		4,492	13.7	80	3.9	8,106	6.4	
**SOCIOECONOMIC STATUS**												
Q1 (lowest quartile)	1,708	15.5	17,114	20.1	<0.001	11,069	33.7	350	17.0	29,541	23.3	<0.001
Q2	2,386	21.7	21,010	24.7		9,112	27.7	428	20.8	32,080	25.3	
Q3	3,012	27.4	21,165	24.9		7,941	24.2	493	24.0	31,625	24.9	
Q4 (highest quartile)	3,851	35.0	25,497	30.0		4,625	14.1	784	38.1	33,189	26.2	
Missing	43	0.4	205	0.2		114	0.3	3	0.2	359	0.3	
**SMOKING^a^**												
Non-smoker	3,561	79.8	28,906	76.0	<0.001	8,431	56.3	1,512	82.3	39,386	70.8	<0.001
Smoker	877	19.6	8,855	23.3		6,158	41.1	324	17.6	15,556	28.0	
Unknown	26	0.6	283	0.7		378	2.5	1	0.1	686	1.2	
**PRE-EXISTING MEDICAL CONDITIONS**												
Hypertension	140	1.3	925	1.1	0.08	327	1.0	28	1.4	1,364	1.1	0.2
Diabetes	27	0.3	210	0.2	0.97	103	0.3	6	0.3	334	0.3	0.8
Asthma	541	4.9	3,881	4.6	0.1	2,134	6.5	82	4.0	6,474	5.1	0.02

a *Routine reporting of maternal smoking on the perinatal record form did not begin until 1998. Therefore, smoking data are unavailable for 71,377 pregnancies occurring before this date*.

There was a significant interaction between age (≤ 35, >35 years) and night shift work (Adjusted β = 0.379, SE = 0.158, *p* = 0.02) in relation to requirement for fertility treatment. As shown in Table 3, among younger women, night shift workers were more likely to require treatment compared to all other women (Adjusted OR = 1.40, 95% CI 1.19–1.64). When the comparison group comprised day workers, results were somewhat attenuated but both the interaction term and the association between night shift work and fertility treatment remained statistically significant. No association was observed among older women. Associations did not change appreciably upon adjustment for ethnicity and socioeconomic status.

**Table 3:** Use of fertility treatment to conceive a first birth among women who work night shift compared to all other women and day workers.

	**Use of fertility treatment**				**Night shift workers vs all other women**		**Night shift workers vs day workers**	
		**Night shift workers**	**All other women**	**Day workers**	**Unadjusted OR [95%CI]**	**Adjusted^a^ OR [95%CI]**	**Unadjusted OR [95%CI]**	**Adjusted^a^ OR [95%CI]**
Women aged ≤ 35 years	*n*	177	1,311	1,065	1.49 [1.28–1.75]	1.40 [1.19–1.64]	1.31 [1.12–1.54]	1.27 [1.08–1.50]
	Total	10,909	111,018	79,242				
	%	1.6	1.2	1.3				
Women aged > 35 years	*n*	66	504	449	0.98 [0.75-1.28]	0.96 [0.74-1.25]	0.92 [0.71–1.21]	0.92 [0.71−1.21]
	Total	910	6,834	5,749				
	%	7.3	7.4	7.8				

*CI, confidence interval; OR, odds ratio*.

a *Analyses adjusted for ethnicity and socio-economic indexes for areas*.

In sensitivity analyses women employed as nurses were excluded. This reduced the sample size available but results indicated a similar pattern of associations, with women's age remaining an important modifier of the effect. For example, when night shift workers were compared with all other women, the adjusted result for those ≤35 years was OR = 1.34, 95% CI 1.00–1.80; when the comparison group was day workers, the adjusted result was OR = 1.22, 95% CI 0.90–1.64.

In the 4-year period in which information on smoking was available, smokers were 60% less likely to have conceived using fertility treatment (consistent with findings for socioeconomic status). Inclusion of smoking in the fully adjusted model did not alter the overall association between night shift work and use of fertility treatment for conception, regardless of the comparison group. For example, when night shift workers were compared with all other women, the adjusted result for those ≤35 years was OR = 1.44, 95% CI 1.08–1.93; when the comparison group was day workers, the adjusted result was OR = 1.32, 95% CI 0.98–1.77.

Table 4 shows the prevalence of infertility diagnoses separately for night shift workers, all other women and day workers. Endometriosis and menstrual irregularity were more common among night shift workers compared to the other two groups (Table 4). Conversely, unexplained infertility was less likely among night shift workers, although these results did not reach statistical significance. There was little difference in the prevalence of ovulatory dysfunction or tubal blockage/problem among the groups.

**Table 4:** Associations between female infertility categories and night shift work among women who required fertility treatment to conceive a first birth.

	**Prevalence of infertility diagnoses n (%)**			**Night shift workers vs all other women**		**Night shift workers vs day workers**	
**Infertility category**	**Night shift workers (*n* = 243)**	**All other women (*n* = 1,815)**	**Non-shift employed workers (*n* = 1,514)**	**Unadjusted OR [95%CI]**	**Adjusted^a^ OR [95%CI]**	**Unadjusted OR [95%CI]**	**Adjusted^a^ OR [95%CI]**
Ovulatory dysfunction	48 (19.8)	379 (20.9)	318 (21.0)	0.93 [0.67–1.30]	0.93 [0.66–1.31]	0.93 [0.66–1.30]	0.90 [0.64–1.27]
Endometriosis	76 (31.3)	451 (24.8)	390 (25.8)	1.37 [1.03–1.84]	1.39 [1.04–1.87]	1.31 [0.98–1.76]	1.34 [1.00–1.80]
Tubal blockage/problem	77 (31.7)	648 (35.7)	520 (34.3)	0.84 [0.63–1.11]	0.82 [0.62–1.10]	0.89 [0.66–1.19]	0.88 [0.65–1.18]
Menstrual irregularity	76 (31.3)	451 (24.8)	366 (24.2)	1.38 [1.03–1.84]	1.38 [1.03–1.85]	1.42 [1.06–1.91]	1.42 [1.05–1.91]
Unexplained infertility	31 (12.8)	307 (16.9)	269 (17.8)	0.72 [0.48–1.07]	0.73 [0.49–1.08]	0.68 [0.45–1.01]	0.69 [0.46–1.03]

*CI, confidence interval; OR, odds ratio*.

a *Analyses adjusted for ethnicity and socio-economic indexes for areas*.

In sensitivity analyses, smoking was assessed as a potential confounder of the associations between night shift work and infertility diagnoses. Interrogation of the restricted dataset showed that smoking would not influence associations between night shift work and either endometriosis or menstrual irregularity, since it was not associated with these diagnoses (hence effect estimates did not change when smoking was including in a multivariate analysis). Body mass index was available for 1,774 women who underwent fertility treatment. The distributions of BMI were similar for all groups with BMI mean[sd] kg/m^2^ for night shift workers 24.9 [5.2] vs. 24.4 [4.9] for day workers (*p* = 0.24) and 24.6 [5.0] for all other women (*p* = 0.46).

## Discussion

We found the association between potential night shift work and use of fertility treatment to conceive a first birth was significantly modified by women's age. Potential night shift work increased the likelihood of fertility treatment in young women up to and including 35 years by an estimated 27–40%, depending on the reference group, but no association was observed among women over 35 years, when compared to day workers. Night shift workers who received fertility treatment were 30–40% more likely to have an infertility diagnosis of endometriosis or menstrual irregularity, and 30% less likely to experience unexplained infertility, compared to other women requiring treatment to achieve a first birth.

To our knowledge, this is the first study to investigate night shift work and use of fertility treatment. Our results are consistent with a population based Danish study which investigated age-standardized differences in female fertility treatment rates across industries, finding that hospital workers—among whom night shift is common—were significantly more likely to undergo fertility treatment than other economically active women (30). Inherent bias may exist among healthcare workers seeking fertility treatment compared to other occupations because of health care workers' increased awareness of the availability, and perhaps perception of need for fertility treatment. While there is some suggestion in the literature that this is the case, the strongest factor predicting fertility awareness is education level (31, 32). Further, sensitivity analysis in the present study showed that the findings also applied to women who worked night shift but were not nurses.

Other studies have investigated infertility (defined in terms of time to conception) among shift workers. In a 2014 meta-analysis of five cohorts, female shift workers had a significantly higher rate of infertility compared to non-shift workers (OR = 1.80, 95% CI 1.01–3.20) (10). Conversely, a later study of 1,739 women in the Nurses' Health Study 3 Cohort found no associations between different shift work patterns and time to conception (33). Similarly, a recent preconception cohort study of 6,873 women found no association between shift work patterns and fecundability (34).

Our results regarding the infertility diagnoses and reproductive conditions among night shift workers are consistent with previous literature on shift work, menstrual irregularity and prolonged time to conception (7, 10), and a smaller literature on shift work and endometriosis (8, 9). In addition, an association between shift work and menstrual irregularity has been demonstrated in studies of different design and among different samples. This includes questionnaire-based studies, where data on menstrual function was collected independently of the clinical infertility treatment setting (7), and studies where nurses did not form the majority of the sample (35).

The more frequent diagnoses of menstrual irregularity and endometriosis among night shift workers requiring fertility treatment are consistent with biological mechanisms associated with night and rotating shift work. Different hormone systems follow different secretory patterns and adapt at different rates to circadian disruption, so night and rotating shift work is likely to produce at least some asynchrony in these systems (36, 37). Circadian activity is coordinated by the suprachiasmatic nucleus in the hypothalamus, which relays information from environmental stimuli to other parts of the brain and peripheral organs (36, 38). Animal studies suggest that optimal functioning of the suprachiasmatic nucleus is required to produce the luteinizing hormone (LH) surge and ensuing ovulation and that melatonin interacts with gonadotropins, including augmentation of the LH surge (36, 39). In this circumstance, perturbation of the LH surge may disrupt the cyclicity of ovulation in women who otherwise do not have anovulatory infertility or poor ovarian reserve. In a prospective study of couples attending a fertility center, women who worked evening/night/rotating shifts had significantly lower oocyte yield following controlled ovarian stimulation compared to day workers, but no difference in measures of ovarian reserve, such as antral follicle count and follicle stimulating hormone (40).

Circadian misalignment and impaired sleep are also associated with neuroendocrine stress (increased cortisol and catecholamine activity), oxidative stress, altered immune function and low-grade system inflammation (41). Impaired immune function and inflammatory responses in night shift workers may contribute to increased susceptibility to endometriosis, as impaired immune surveillance and reactive oxygen species have been implicated in the inflammatory and pathophysiological processes of the disease (42–44).

Individuals have been shown to vary in their ability to tolerate night shift work. Those with poor tolerance experience symptoms such as gastrointestinal disturbance, sleep disturbance, fatigue, and changes in mood (irritability, low affect) and behavior (45, 46). Thus, self-selection into or out of shift schedules is probable (47). It is possible night shift workers who required fertility treatment for a first birth had relatively poor tolerance for shift work, but limited choice about the matter, for example, as occurs in more junior nursing roles. In a systematic review of individual differences in tolerance to shift work (48), evidence regarding age and tolerance of shift work was mixed, but few studies were conducted among female workers and even fewer considered women aged under 30 years.

The elevated use of fertility treatment among night shift workers was magnified when the comparison group comprised all primiparous women, including those not in paid employment. The group of women who reported being engaged in home duties, in particular, was larger than expected for primiparous women. The great majority of these women had their first birth at less than age 30 years and were relatively disadvantaged, suggesting that any paid work they had prior to pregnancy may have been low skilled, lacked paid maternity leave, and was not seen as a career. Hence a degree of non-reporting of former occupation is likely among such women (15). It is difficult to gauge whether misclassification bias could arise from this source, but some reassurance is provided by the fact that assisted conception occurred in 1.7% of women reporting home duties, similar to the proportion for women in paid employment who did not work night shift (1.8%).

Strengths of this study are the large, population-based cohort of over 128,000 primiparous women, and the detailed health information available for women undergoing fertility treatment. Restriction of the analysis to primiparous women substantially addresses any bias due to the infertile worker effect, whereby childless women are more likely to remain in the workforce (24). The JEM used was developed in a representative population of women of the same nationality and contemporary to the study population. In a validation study, the JEM performed almost as well as job specific questionnaires in terms of reproducing an established association (18). JEMs are a well-accepted and commonly-used method to extrapolate exposure from occupational data where direct measurements cannot be made (49, 50). A further strength of a JEM is that it is applied consistently to all study participants, attenuating observation bias or at least rendering it non-differential.

The use of JEMs to classify exposure has limitations. JEMs classify exposure at the occupation-level rather than the individual-level. No information was available on the actual night shift exposure of individual women. There is therefore likely to be exposure misclassification. However, as misclassification occurs independently of outcome status (i.e., non-differentially), this would tend to move estimates toward the null.

It is also important to note that women who access fertility treatment may not be representative of all infertile or subfertile women, particularly in terms of socioeconomic status. Residual confounding may also be present, as due to the nature of the registry data, we are unable to consider other potential confounders such as diet, education level and working hours. A further limitation of this study is that we do not have information on fertility treatment for treated women who did not conceive or whose gestation did not reach 20 weeks (51), and we do not know if our findings are affected by survivorship bias. We also do not have information on menstrual irregularity or endometriosis among women who conceived naturally. Lastly, given the timeframe of the data, there may have been changes to working conditions and the accessibility of fertility treatment over time that may alter the associations observed in this study. Alternatively, the advantage of an older data set is that the average age of first birth was younger (and more women attempted parenthood at a younger age) and there was more reluctance to undergo treatment.

In conclusion, this study adds to literature implicating night shift work in reproductive health problems (4, 7, 10). Adverse effects appeared in women <35 years only, who may represent a vulnerable subgroup with poor tolerance of the sequelae of night shift work, and this deserves further research. Providing these women with a degree of control and choice about shift schedule may be the best way to enable them to maintain income and career and health, while accommodating shift work (52). Other strategies to mitigate circadian disruption exist, for example tailored sleep plans (53); these should be promoted and further practical avenues explored.

## Data Availability Statement

The data analyzed in this study is subject to the following licenses/restrictions: The authors do not have permission to share the data as they were provided specifically for the scope of research as approved by the ethics committees. Requests to access these datasets should be directed to https://www.santdatalink.org.au/.

## Ethics Statement

The studies involving human participants were reviewed and approved by South Australian Department of Health Human Research Ethics Committee, University of Adelaide Human Research Ethics Committee and Flinders University Human Research Ethics Committee. Written informed consent for participation was not required for this study in accordance with the national legislation and the institutional requirements.

## Author Contributions

RF participated in study design, planned and conducted the statistical analysis, interpreted the results, and drafted the manuscript. VM, JM, MW, and MD participated in study design, interpretation of results, critically reviewed, and contributed to the manuscript. All authors contributed to manuscript revision, read, and approved the submitted version.

## Conflict of Interest

The authors declare that the research was conducted in the absence of any commercial or financial relationships that could be construed as a potential conflict of interest.
